# Use of Social Media by Western European Hospitals: Longitudinal Study

**DOI:** 10.2196/jmir.1992

**Published:** 2012-05-01

**Authors:** Tom H Van de Belt, Sivera AA Berben, Melvin Samsom, Lucien JLPG Engelen, Lisette Schoonhoven

**Affiliations:** ^1^Radboud REshape and Innovation CentreRadboud University Nijmegen Medical CentreNijmegenNetherlands; ^2^Emergency Healthcare NetworkRadboud University Nijmegen Medical CentreNijmegenNetherlands; ^3^Executive BoardRadboud University Nijmegen Medical CentreNijmegenNetherlands; ^4^Scientific Institute for Quality of HealthcareRadboud University Nijmegen Medical CentreNijmegenNetherlands; ^5^Faculty of Health SciencesUniversity of SouthamptonSouthamptonUnited Kingdom

**Keywords:** Social media, health 2.0, medicine 2.0, eHealth, participatory health care, patient empowerment, Web 2.0, patient-centered care

## Abstract

**Background:**

Patients increasingly use social media to communicate. Their stories could support quality improvements in participatory health care and could support patient-centered care. Active use of social media by health care institutions could also speed up communication and information provision to patients and their families, thus increasing quality even more. Hospitals seem to be becoming aware of the benefits social media could offer. Data from the United States show that hospitals increasingly use social media, but it is unknown whether and how Western European hospitals use social media.

**Objective:**

To identify to what extent Western European hospitals use social media.

**Methods:**

In this longitudinal study, we explored the use of social media by hospitals in 12 Western European countries through an Internet search. We collected data for each country during the following three time periods: April to August 2009, August to December 2010, and April to July 2011.

**Results:**

We included 873 hospitals from 12 Western European countries, of which 732 were general hospitals and 141 were university hospitals. The number of included hospitals per country ranged from 6 in Luxembourg to 347 in Germany. We found hospitals using social media in all countries. The use of social media increased significantly over time, especially for YouTube (n = 19, 2% to n = 172, 19.7%), LinkedIn (n =179, 20.5% to n = 278, 31.8%), and Facebook (n = 85, 10% to n = 585, 67.0%). Differences in social media usage between the included countries were significant.

**Conclusions:**

Social media awareness in Western European hospitals is growing, as well as its use. Social media usage differs significantly between countries. Except for the Netherlands and the United Kingdom, the group of hospitals that is using social media remains small. Usage of LinkedIn for recruitment shows the awareness of the potential of social media. Future research is needed to investigate how social media lead to improved health care.

## Introduction

Social media are defined as a group of Internet-based applications that build on the ideological and technological foundations of Web 2.0, and they allow the creation and exchange of user-generated content [1]. Social media allow individuals to participate in online social networks and turn communication into interactive dialogue, using highly accessible and scalable communication applications [2]. Of all young Internet users (18–24 years of age) in the European Union, 80% use social media [3]. In the Netherlands, this percentage is even higher, with 91% using social media [3].

Facebook and Twitter are well-known examples of social media, which have become mainstream social technologies [4]. Facebook has over 800 million active users [5]. For comparison, the United States has 310 million inhabitants [6]. One of the success factors of social media is that many are free of charge. Social media play an increasingly important role in our society, and they are being used for a large variety of purposes, varying from finding a job or an employee to finding a partner or planning a trip. Also, a growing number of people are using mobile devices such as smartphones and tablet computers, which allow them to use social media from any place, at any time [1].

Social media empower users by allowing them to communicate effectively and have access to all kinds of information. Not only individuals use social media; companies use them too. It helps them to listen better to customers to hear what they want. Barnes and Mattson studied use of blogs and Twitter by the 500 largest corporations in the United States [7]. They found a steady adoption of blogs and an explosive growth of Twitter. As these companies have great influence on the commercial sector, it is expected that social media will become more important in the business world.

In health care, patients increasingly use social media to communicate and share information. This is one of the fundamentals of what is described as Health 2.0 or Medicine 2.0 [8]. Patients share their stories and information on social media, which are rapidly indexed by search engines like Google and can be found easily. Seeing that many patients start by performing a Google search, it seems relevant for hospital organizations to be active on social media. For example, 64% of all respondents of an online questionnaire among patients in the United States start by performing a search to analyze their condition [9]. Another reason why hospital organizations should embrace social media is that it may contribute to quality improvements in health care. Active use of social media not only speeds up communication and improves information provision for patients; it allows caregivers to engage patients in the delivery of care, and for caregivers and patients to make decisions collaboratively and improve their relationship [10]. In this way, using social media improves patient-centered care [11].

There are also beneficial aspects for the hospital organization itself. Several studies reported that social media can improve communication among staff, facilitate networking, attract visitors to the hospital’s website, build the hospital’s brand, and be used for recruitment for research projects [12,13].

A descriptive study performed in the United Kingdom found that 40% of the 152 health care organizations they studied used one or more types of social media, but that there was little interaction with online visitors (eg, patients) [14]. Also, many organizations were simply “seeding” information. In the United States, the use of social media by hospitals has been noted. Bennett documented that 674 hospitals had a Twitter account and 448 were on YouTube [15]. Considering that the United States has a total of 5000 hospitals, around 15.7% of all hospitals in the United States are on Twitter, 20.3% are on Facebook, and 10.9% are on YouTube [16]. However, it is unknown whether and how Western European hospitals use social media. Therefore, the target of this study was to identify the extent to which European hospitals use social media.

## Methods

In this longitudinal study, we explored the use of social media by hospitals in 12 Western European countries through an Internet search.

### Inclusion Criteria

We included the following Western European countries: the Netherlands, Belgium, Luxembourg, Germany, Austria, Switzerland, the United Kingdom, Ireland, Norway, Sweden, Finland, and Denmark. To retrieve a comprehensive list of hospitals for each country, we searched for lists of hospitals with detailed information on Wikipedia and the Hospitals Worldwide website [17,18]. Second, we contacted colleagues from the included countries and asked for official lists of hospitals. Third, we consulted country-specific websites with detailed information. Fourth and last, we used Google and each hospital’s website to find additional information such as contact information or the number of beds. We included only hospitals with a website and at least 200 beds. If hospitals were part of a larger hospital organization with a central website, we explored the central website only and counted these hospitals as 1 hospital.

### Variables

For each hospital we recorded the following characteristics: official name, address, country, province or state, email, number of beds, and number of hospitals included in the organization.

Since no scientific evidence was available on the popularity of different social media, we used information from websites and infographics to decide which social media were most popular and needed to be included in the study [19,20]. We gathered data about the following social media: YouTube, Twitter, Facebook, LinkedIn, and blogs (weblogs). We defined blog by the presence of the following characteristics: reverse chronological order of publication, regular updates (>1 per month), and the possibility to post comments. Facebook has different types of pages. In this study, we distinguished between company pages and group pages. For each medium, we searched for relevant data on use such as the number of friends or followers, the number of videos or tweets, and the date of registration. For each medium, we recorded whether the media could be found via the hospital’s website.

### Data Collection

Between April 2009 and July 2011, we collected data for each country during the following three time periods: T1 (April to August 2009), T2 (August to December 2010), and T3 (April to July 2011). YouTube accounts, Twitter accounts, and blogs were measured at T1, T2, and T3. For Facebook and LinkedIn, we performed two measurements, at T2 and T3.

Two researchers collected the data. A predefined search protocol was used, containing a 3-step search strategy. First, we visited the hospital’s website and searched for social media. We also used the website’s search function (if available). Second, we searched for the hospital’s name within the different types of social media such as YouTube. Third and last, we used Google for more specific search queries, such as the hospital’s name and *Twitter*. Table 1 presents the search protocol. Before the official start, the two researchers involved in the search discussed the results for 20 hospitals. Since all variables in this study are unambiguous (eg, number of beds, Twitter account: “yes” or “no”), no relevant differences or issues appeared.

**Table 1:** Search protocol for data collection.

Step	Protocol
1	Select hospital from list.
2	Visit official website and add contact information to table. Find using standard search tool (ie, Google).
3	Record number of beds (total). Include hospitals with >200 beds.
4	If included, proceed to next steps.
5	Add general information.
6	Look for different types of social media on hospital’s website and add to table.
7	Use search option on hospital’s website and search the terms *YouTube*, *movie*, *film*, *Twitter*, *Facebook*, *blog*, *LinkedIn*, and *weblog*. Add all new social media to the table.
8	Visit Twitter.com, Youtube.com, Facebook.com, and LinkedIn.com and search on hospital’s official name. Add all new social media to the table.
9	Use specific search queries in Google, eg, the hospital’s name AND *Facebook*. Add all new social media to the table.
10	Add other relevant information for all types of social media, eg, number of friends and followers, date of registration.

### Data Validation

We contacted all organizations with the request to validate the results for their hospital. We sent emails to each hospital’s general email address as stated on their official website, most likely on the Contact page. The email contained a description of this study by the Radboud REshape & Innovation Center, University Nijmegen Medical Centre, and a unique link to an online database. Receivers were able to make changes or add information or comments. We sent 873 email requests. Of these, 45 messages (5%) were returned as undeliverable, and 44 hospitals validated the results (5%).

### Analysis

We used descriptive statistics to describe the basic features of our data and the use of social media by the included hospitals. We calculated percentages, means, and standard deviations for normally distributed data, and medians and interquartile ranges for nonnormally distributed data. Cochran Q test was used to analyze the differences in social media usage between the three measurements within individual countries. In case of significant differences, we used the McNemar test for post hoc testing. Furthermore, we analyzed the differences in social media usage between countries at T3 by using the chi-square test. Finally, we used the Wilcoxon rank test to analyze the nonnormally distributed data for number of videos, views, and followers between T2 and T3 within the included countries.

## Results

In total we looked at 873 hospitals from 12 Western European countries: 732 general hospitals and 141 university hospitals. The number of included hospitals per country ranged from 6 in Luxembourg to 347 in Germany. The mean number of beds per hospital was 544. Table 2 presents general characteristics of the hospitals.

**Table 2:** Hospitals included in the analysis and their general details.

Country^a^	Number of hospitals			Number of beds, mean (SD)
	Total	General hospitals	University hospitals	
NL	88	80	8	549 (278)
BE	91	79	12	450 (261)
LU	6	5	1	363 (139)
DE	347	314	33	533 (445)
AT	25	19	6	775 (587)
CH	41	39	2	389 (232)
UK	175	123	52	624 (282)
IR	28	21	7	392 (192)
NO	17	11	6	480 (238)
SE	22	17	5	698 (511)
FI	9	7	2	697 (544)
DK	24	17	7	551 (286)
Total	873	732	141	544 (376)

^a ^NL = the Netherlands, BE = Belgium, LU = Luxembourg, DE = Germany, AT = Austria, CH = Switzerland, UK = United Kingdom, IR = Ireland, NO = Norway, SE = Sweden, FI = Finland, DK = Denmark.

We found hospitals using social media in all countries. The use of social media increased over time, and we found significant differences between countries. Table 3, Table 4, and Table 5 show the results by country.

**Table 3:** Social media usage (T1–T3)^a ^in 12 Western European countries (YouTube and Twitter).

Country^b^	YouTube, n (%)			*P *value		Twitter, n (%)			*P *value	
	T1	T2	T3	T1 vs T2 vs T3^c^	T1 vs T3^d^	T1	T2	T3	T1 vs T2 vs T3^c^	T1 vs T3^d^
NL (n = 88)	9 (10%)	23 (26%)	33 (38%)	<.001	<.001	4 (5%)	27 (31%)	49 (56%)	<.001	<.001
BE (n = 91)	1 (1%)	4 (4%)	5 (5%)	.04	.14	0	2 (2%)	6 (7%)	.009	.03
LU (n = 6)	0.0	1 (17%)	1 (17%)	.37	1	0	0	0	ND^e^	ND
DE (n = 347)	3 (1%)	20 (6%)	52 (15%)	<.001	<.000	2 (1%)	9 (3%)	23 (7%)	<.001	<.001
AT (n = 25)	0	3 (12%)	3 (12%)	.05	.25	0	0	0	ND	ND
CH (n = 41)	0	2 (5%)	5 (12%	.02	.06	0	1 (2%)	1 (2%)	.37	1
UK (n = 175)	6 (3%)	37 (21%)	62 (35%)	<.001	<.001	4 (2%)	42 (24%)	68 (39%)	<.001	<.001
IR (n = 28)	0	0	0	ND	ND	0	0	1 (4%)	.37	1
NO (n = 17)	0.0	2 (12%)	3 (18%)	.1	1	0	2 (12%)	8 (47%)	.002	.008
SE (n = 22)	0	5 (23%)	5 (23%)	.007	1	0	2 (9%)	2 (9%)	.14	.5
FI (n = 9)	0	0	0	ND	ND	0	0	0	ND	ND
DK (n = 24)	0.0	2 (8%)	3 (13%)	.1	.25	0	0	0	ND	ND
All (n = 873)	19 (2%)	99 (11%)	172 (19.7%)	<.001	<.001	10 (1%)	85 (10%)	158 (18.1%)	<.001	<.001

^a ^T1 = April to August 2009, T2 = August to December 2010, T3 = April to July 2011.

^b ^NL = the Netherlands, BE = Belgium, LU = Luxembourg, DE = Germany, AT = Austria, CH = Switzerland, UK = United Kingdom, IR = Ireland, NO = Norway, SE = Sweden, FI = Finland, DK = Denmark.

^c ^Cochran Q test (df = 2).

^d ^McNemar test (df = 2).

^e ^No data.

**Table 4:** Social media usage (T1–T3)^a ^in 12 Western European countries (Facebook, blogs, and LinkedIn).

Country^b^	Facebook, n (%)			*P *value	Blog, n (%)			*P *value		LinkedIn, n (%)			*P *value
	T1	T2	T3	T2 vs T3^c^	T1	T2	T3	T1 vs T2 vs T3^d^	T1 vs T3^c^	T1	T2	T3	T2 vs T3^c^
NL (n = 88)	ND^e^	0	13 (15%)	<.001	2 (2%)	5 (6%)	4 (5%)	.1	.5	ND	48 (55%)	71 (81%)	<.001
BE (n = 91)	ND	20 (22%)	62 (68%)	<.001	2 (2%)	2 (2%)	2 (2%)	1	1	ND	20 (22%)	41 (45%)	<.001
LU (n = 6)	ND	0	3 (50%)	.25	0	0	0	ND	ND	ND	0	2 (33%)	.5
DE (n = 347)	ND	26 (8%)	232 (66.9%)	<.001	0	0	1 (1%)	.37	1	ND	6 (2%)	10 (3%)	.22
AT (n = 25)	ND	1 (4%)	21 (84%)	<.001	0	0	0	ND	ND	ND	1 (4%)	3 (12%)	.5
CH (n = 41)	ND	4 (10%)	15 (37%)	.001	0	0	0	ND	ND	ND	5 (12%)	9 (22%)	.13
UK (n = 175)	ND	31 (18%)	163 (93.1%)	<.001	0	10 (6%)	12 (7%)	<.001	<.001	ND	71 (41%)	97 (55%)	<.001
IR (n = 28)	ND	0	23 (82%)	<.001	0	0	0	ND	ND	ND	0	3 (11%)	.25
NO (n = 17)	ND	2 (12%)	15 (88%)	<.001	0	0	1 (6%)	.37	1	ND	8 (47%)	13 (76%)	.06
SE (n = 22)	ND	0	10 (45%)	<.001	3 (14%)	3 (14%)	2 (9%)	.37	1	ND	15 (68%)	17 (77%)	.5
FI (n = 9)	ND	0.0	7 (78%)	.02	0	0	0	ND	ND	ND	0	1 (11%)	1
DK (n = 24)	ND	1 (4%)	21 (88%)	<.001	0	0	1 (4%)	.37	1	ND	5 (21%)	11 (46%)	.03
All (n = 873)	ND	85 (10%)	585 (67.0%)	<.001	7 (1%)	20 (2%)	23 (3%)	<.001	<.001	ND	179 (20.5%)	278 (31.8%)	<.001

^a ^T1 = April to August 2009, T2 = August to December 2010, T3 = April to July 2011.

^b ^NL = the Netherlands, BE = Belgium, LU = Luxembourg, DE = Germany, AT = Austria, CH = Switzerland, UK = United Kingdom, IR = Ireland, NO = Norway, SE = Sweden, FI = Finland, DK = Denmark.

^c ^McNemar test (df = 2).

^d ^Cochran Q test (df = 2).

^e ^No data.

**Table 5:** YouTube videos, views, and Twitter followers at T2 and T3^a^.

Country^b^	YouTube videos per account, median (IQR^c^)		*P *value	YouTube views per account, median (IQR)		*P *value	Twitter followers per account, median (IQR)		*P *value
	T2	T3	T2 vs T3^d^	T2	T3	T2 vs T3^d^	T2	T3	T2 vs T3^d^
NL (n = 88)	5 (2–20)	9 (5–26)	.03	839 (221–1721)	4828 (976–12022)	<.001	119 (48–235)	336 (150–748)	<.001
BE (n = 91)	3 (2–3)	7 (3–9)	.18	241 (145–241)	6648 (3332–13241)	.04	175	127 (41–232)	ND^e^
LU (n = 6)	5 (a)	4 (a)	ND	141	244	ND	0	0	ND
DE (n = 347)	2 (2–17)	6 (3–19)	01	1809 (737–27,823)	1920 (382–11366)	.001	51 (27–76)	90 (30–309)	.18
AT (n = 25)	20 (10–22)	32 (18–58)	.11	10,930 (5465–12,755)	26,251 (19,855–29,692)	.18	0	0	ND
CH (n = 41)	2 (2–3)	6 (3–16)	1	3 (a)	3717 (2003–3853)	ND	19	63	ND
UK (n = 175)	5 (2–8)	7 (4–16)	.004	256 (137–1436)	2372 (880–7313)	<.0001	311 (135–625)	464 (145–1019)	<.001
IR (n = 28)	0	0	ND	0	0	ND	0	44 (a)	ND

NO (n = 17)	7 (5–8)	4 (3–8)	.32	2962 (2700–3223)	5250 (5200–7082)	.18	57 (30–83)	200 (65–370)	.18
SE (n = 22)	13 (7–16)	12 (4–12)	.13	560 (458–7199)	3146 (1892–12029)	.35	84 (75–92)	142 (116–169)	.18
FI (n = 9)	0	0	ND	0	0	ND	0	0	ND
DK (n = 24)	1 (1–2)	3 (3–3)	ND	101 (51–152)	120.0 (71–168)	ND	0	0	ND
All (n = 873)	4 (2–13)	7 (3–16)	<.001	575 (190–2444)	3074 (724–10110)	<.001	204 74–579)	271 (85–724)	<.001

^a ^T1 = April to August 2009, T3 = April to July 2011.

^b ^NL = the Netherlands, BE = Belgium, LU = Luxembourg, DE = Germany, AT = Austria, CH = Switzerland, UK = United Kingdom, IR = Ireland, NO = Norway, SE = Sweden, FI = Finland, DK = Denmark.

^c ^Interquartile range.

^d ^Wilcoxon signed rank test.

^e ^No data.

### YouTube

YouTube accounts were found in 10 countries (Table 3). At T3, we found significant differences in the percentage of YouTube usage (χ^2^_11 = 73.9,_ *P *< .001). The Netherlands (38%, n = 33) and the United Kingdom (35%, n = 62) had the highest percentage of hospitals with a YouTube account. During the research period, the percentage of YouTube accounts increased significantly (Table 3). The median number of videos per YouTube account at T3 was 7 (Table 5).

### Twitter

Twitter accounts were found in 8 of 12 countries (Table 3), with significant differences between countries (χ^2^_11 = 209.2,_ *P *< .001) at T3. The Netherlands (56%, n = 49), the United Kingdom (39%, n = 68), and Norway (47%, n = 8) had the highest percentages of hospitals with a Twitter account. The median number of followers for all countries at T3 was 271 (Table 5). We identified 1 hospital with 3300 followers.

### Facebook

**Figure 1:**
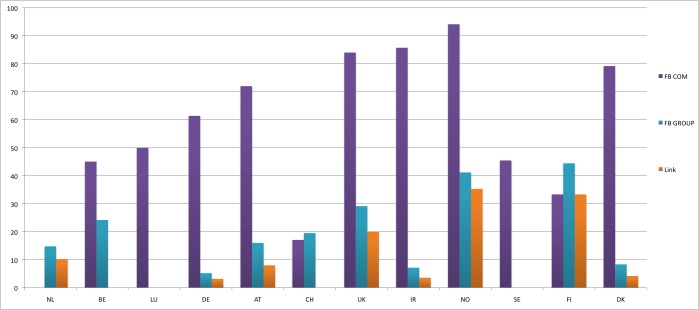
Percentage of Facebook company profiles (FB COM), group pages (FB GROUP), and links (Link) to a Facebook account on hospital websites at T3 (April to July 2011). NL = the Netherlands, BE = Belgium, LU = Luxembourg, DE = Germany, AT = Austria, CH = Switzerland, UK = United Kingdom, IR = Ireland, NO = Norway, SE = Sweden, FI = Finland, DK = Denmark.

Facebook accounts were found in all countries, ranging from 15% (n = 13) in the Netherlands to 93.1% (n = 163) in the United Kingdom (Table 4). At T3, there was a significant difference between all countries in the percentage of Facebook usage (χ^2^_11 = 202.1,_ *P *< .001). Facebook usage increased significantly in 11 countries. Two types of Facebook accounts were found: company profiles and group pages (Figure 1). The number of Facebook group pages was lower, ranging from 0% in Luxembourg to over 40% in Finland and Norway. Apart from 2 countries (Norway and Finland), having a Facebook page accessible through the hospital’s website was an exception (Figure 1).

### Blogs

Blogs were found in 7 of the 12 countries, ranging from 1% (n = 1) in Germany to 9% (n = 2 ) in Sweden (Table 4). We found blogs less frequently than the other types of social media. The percentages of blogs differed significantly between countries (χ^2^_11 = 28.5,_ *P *= .003).

### LinkedIn

We measured LinkedIn during two periods (T2 and T3). We found significantly increased usage in 4 countries. At T3, the percentage of LinkedIn accounts ranged from 3% (n = 10) in Germany to 81% (n = 71) in the Netherlands (Table 4), and the percentages were significantly different (χ^2^_11 = 336.4,_ *P *< .001). Of all 873 hospitals, we found 1 hospital with a link to their LinkedIn profile on their website.

## Discussion

In this longitudinal study we explored the use of social media by 873 hospitals in 12 Western European countries. The use of social media increased in all of the countries, especially YouTube (from 2% to 19.7%), LinkedIn (20.5% to 31.8%), and Facebook (10% to 67.0%). This increased use of social media has been confirmed by other studies [14]. Interestingly, the use of Twitter, Facebook, and YouTube in Europe appeared to be higher than in the United States [15].

There are notable differences between the 12 countries. The use of Twitter was especially popular in the United Kingdom, the Netherlands, and Norway. At the third measurement, almost half of all hospitals in the Netherlands and in Norway were on Twitter. YouTube was used by 35% of the hospitals in the United Kingdom and 38% in the Netherlands, whereas the use of YouTube varied from 0% to 23% in all other countries. There are several possible reasons for the differences between countries that we found. First, the use of social media could be related to the Internet penetration in a specific country. However, the differences in broadband penetration in Europe are small [21]. Second, there may be an influence of local or country-specific social media. An example is Hyves, which was, until recently, the most popular social network in the Netherlands, with more than 11 million members [22]. This could explain why Facebook was less popular in the Netherlands than in other countries. It is difficult to predict the popularity or influence of other social media. Online sources show that Facebook, when Hyves is excluded, was the most popular social media network in all other countries during the research period [19,20].

The activity of hospitals on social media increased during the research period, as the number of videos and viewers of YouTube channels, and of Twitter followers increased. Furthermore, the increased usage of LinkedIn was notable in the Netherlands and the United Kingdom at the third measurement. Hospitals in these countries seem to be aware of the benefits of recruiting personnel that LinkedIn offers. However, the observation that only 5% (n = 48) of all 873 hospitals had a link to their YouTube channel and 10% (n = 90) had a link to their Twitter feed on their website indicates that hospitals are not using the full potential of all types of social media yet. Based on this study, we cannot say anything about the content of videos, tweets, and messages. However, our data show that an ever-increasing number of users are watching the videos and reading the tweets.

Since Western European hospitals have become aware of social media and increasingly use it, we foresee great opportunities to improve health care and to stimulate participatory health care. Various studies have described improvements that social media could offer to health care, such as greater transparency, openness, and communication, and improved patient support and knowledge translation [4,10]. Therefore, research should be focused on describing best practices, which may help speed up implementation of social media. Furthermore, it would be worthwhile to identify for what purposes hospitals use social media and to what extent social media improve participatory health care. For a complete overview, future research should also focus on the challenges and risks of using social media, such as legal constraints, fraud, and budget constraints. These topics are also important research subjects in the light of the discussion about desirability of social media usage by health care professionals.

Our study has some limitations that need to be discussed. In a few cases, we experienced difficulties determining whether a social network was official (was initiated and maintained by the hospital itself). However, we gave hospitals the opportunity to correct their data. Another aspect is the differences between health care systems in the included countries. We found that in a few countries, some hospital organizations included more than 1 hospital. Since we counted these organizations as 1 hospital, our data do not reflect the results of individual hospitals in every country.

Another aspect is that we measured Facebook and LinkedIn only at T2 and T3. It would have been interesting to see the results for T1. However, at the start of the project, we were not aware of hospitals using Facebook or LinkedIn. Since Facebook and LinkedIn became increasingly popular in 2009 and 2010, we decided to include them in the search we conducted in this study.

Awareness and use of social media is growing in Western European hospitals. Social media usage differs significantly between countries. Except for the Netherlands and the United Kingdom, the group of hospitals that are using social media remains small. Usage of LinkedIn for recruitment of personnel shows that hospitals are aware of the potential of social media. Future research is needed to investigate how social media lead to improved health care.
